# The impact of family parenting styles on behavioral and emotional problems in children with attention-deficit/hyperactivity disorder: the mediating role of mobile phone addiction

**DOI:** 10.3389/fpsyg.2026.1729300

**Published:** 2026-03-04

**Authors:** Fang Sun, Ying Wang

**Affiliations:** Department of Child Health Care, Linhai Maternal and Child Health Hospital, Zhejiang, China

**Keywords:** attention deficit disorder with hyperactivity, emotional regulation, internet addiction disorder, parenting, problem behavior

## Abstract

**Objective:**

This study examined the relationships between parenting styles and behavioral-emotional problems in children with Attention-Deficit/Hyperactivity Disorder (ADHD), investigating mobile phone addiction as a potential mediating mechanism.

**Methods:**

A cross-sectional design was employed with 232 children diagnosed with ADHD (aged 6–17 years) and their parents. Participants completed the Swanson, Nolan, and Pelham-IV (SNAP-IV) Rating Scale for ADHD symptoms, the Mobile Phone Addiction Self-Rating Scale, the Child Behavior Checklist (CBCL) Brief Form, and the Egna Minnen Beträffande Uppfostran (EMBU) Parenting Style Scale. Mediation analyses were conducted using Hayes’ PROCESS macro with 5,000 bootstrap samples.

**Results:**

Negative parenting styles (rejection, overprotection) were significantly associated with increased behavioral-emotional problems (*r* = 0.41–0.56, *p* < 0.001), while positive parenting (emotional warmth) showed negative correlations (*r* = −0.38 to −0.36, *p* < 0.001). Mobile phone addiction partially mediated these relationships. The indirect effects were significant (negative parenting: ab = 0.22, 95% CI [0.14, 0.31], accounting for 32.4% of the total effect; positive parenting: ab = −0.16, 95% CI [−0.24, −0.09], accounting for 27.6% of the total effect).

**Conclusion:**

Mobile phone addiction serves as a significant mediator between parenting styles and behavioral-emotional problems in ADHD children. Interventions targeting both parenting practices and digital device management may be beneficial for improving outcomes in this population.

## 1 Introduction

Attention-Deficit/Hyperactivity Disorder (ADHD) represents one of the most prevalent neurodevelopmental disorders in childhood, affecting approximately 5% of school-aged children worldwide (Baker et al., 2020). The disorder is characterized by persistent patterns of inattention, hyperactivity, and impulsivity that significantly impair daily functioning across multiple domains (The American Psychiatric Association, 2022). Children with ADHD frequently experience comorbid behavioral and emotional problems, including oppositional defiant disorder, conduct problems, anxiety, and depression, which compound the challenges faced by affected families (Biederman et al., 2012). A recent meta-analyse has demonstrated that children with ADHD exhibit significantly higher rates of behavioral and emotional dysregulation compared to typically developing peers (Park et al., 2017). The relationship between ADHD symptoms and associated psychopathology is complex and bidirectional. Negative parenting practices, particularly criticism and rejection, have been consistently associated with more severe ADHD symptoms and increased behavioral problems (Cueli et al., 2024). Moreover, the coercive parent–child interaction cycle that often develops in families with ADHD children can perpetuate and exacerbate both core symptoms and associated behavioral difficulties (Samimy, 2022). The Child Behavior Checklist (CBCL) is a valuable tool for identifying children at risk for ADHD. This utility is demonstrated in research, such as the study by Biederman et al. (2021), which utilized the CBCL and found that a significant majority (76%) of youths identified with elevated attention problems on this measure exhibited complex clinical profiles, thereby affirming the CBCL’s effectiveness in pinpointing a population with significant clinical needs.

Parenting styles represent relatively stable patterns of attitudes and behaviors that parents exhibit during child-rearing, encompassing dimensions of emotional warmth, control, and responsiveness (Arrindell et al., 1999). According to interpersonal acceptance-rejection theory and family systems theory, parenting styles exert profound influences on child development and psychological adjustment (Rohner and Lansford, 2017). Three primary dimensions of parenting have been identified as particularly relevant: emotional warmth (characterized by acceptance, support, and affection), rejection (involving criticism, hostility, and neglect), and overprotection (excessive control and intrusion) (Peng et al., 2021). The mechanisms through which parenting styles influence child mental health are multifaceted. Positive parenting characterized by emotional warmth has been shown to foster secure attachment, self-esteem, and emotional regulation skills, serving as a protective factor against psychopathology (Gao et al., 2024). Conversely, negative parenting practices including rejection and overprotection have been linked to increased internalizing and externalizing problems through their impact on children’s basic psychological needs satisfaction (Chen et al., 2025). In the context of ADHD, given that the core symptoms of childhood ADHD—such as impulsivity, inattention, and hyperactivity—directly challenge parental patience and effective disciplinary capacity, this frequently leads to heightened stress and more negative interaction cycles. Consequently, the overall family atmosphere is affected, and the influence of parenting styles may prove particularly pronounced at such times (Deault, 2010). Drawing from family systems and social learning theories, parenting styles are thus conceptualized as a stable, foundational antecedent within the family environment, shaping child outcomes rather than being primarily a reactive factor in the ADHD-symptom cycle (Rohner and Lansford, 2017).

Recent studies have begun to elucidate the pathways through which parenting influences outcomes in children with ADHD. Parent training interventions, which focus on modifying parenting practices, have demonstrated effectiveness in reducing both ADHD symptoms and associated behavioral problems, suggesting a causal role for parenting in symptom expression (Paiva et al., 2024). However, the mechanisms underlying these effects remain incompletely understood, highlighting the need for research examining potential mediating factors (Daley et al., 2014).

The persistence of ADHD symptoms into adolescence brings additional challenges, particularly in the digital age. Adolescents with ADHD are at significantly increased risk for problematic technology use, including smartphone addiction and internet gaming disorder (IGD). Studies indicate high rates of such issues in youth populations (Mohd Salleh Sahimi et al., 2022), with ADHD being a common comorbidity in IGD (Yen et al., 2017). This elevated risk stems from core ADHD symptoms—impulsivity and attention deficits heighten susceptibility to digital rewards, while executive dysfunction impairs self-regulation (Yen et al., 2017; Zhang et al., 2021). Environmental factors, such as parental phubbing, further exacerbate risk by increasing social anxiety and reducing self-esteem (Zhang et al., 2021).

The ubiquity of smartphones has fundamentally altered the developmental landscape, making mobile phone addiction a significant global concern (Sahu et al., 2019), affecting 6–16% of adolescents (Sohn et al., 2019). This addiction is characterized by excessive use, preoccupation, and an inability to control usage despite negative consequences. Its relationship with mental health is bidirectional and complex, forming a vicious cycle where pre-existing psychopathology (such as ADHD) and excessive phone use can mutually reinforce dysfunction (Yuan et al., 2021). Theoretical frameworks help explain this dynamic. Social cognitive theory suggests that environmental factors (including parenting) and individual characteristics (such as ADHD symptoms) interact to shape technology use behaviors (Chen et al., 2025). Furthermore, the compensatory internet use theory posits that individuals may turn to digital devices to cope with psychological distress or fulfill unmet offline needs (Mi et al., 2023). This may be particularly relevant for children with ADHD who often experience social difficulties and academic challenges.

Building upon the interpersonal acceptance-rejection theory (Rohner and Lansford, 2017), negative parenting (e.g., rejection, overprotection) is posited to influence child outcomes by frustrating basic psychological needs for relatedness and autonomy. Within the digital context, compensatory internet use theory (Mi et al., 2023) further suggests that children may turn to excessive smartphone use to cope with this unmet psychological need or to escape family stress. This pathway is theorized to be particularly salient for children with ADHD, whose core deficits in self-regulation and reward sensitivity amplify vulnerability to maladaptive digital engagement.

Emerging evidence suggests that parenting styles significantly influence children’s technology use patterns. Parental phubbing (phone snubbing) and technoference have been associated with increased risk of mobile phone addiction in youth, potentially through their impact on parent–child attachment and children’s emotional regulation (Zhang et al., 2025). Moreover, broader parental attitudes, such as rejection, also play a critical role. For example, a recent study found that perceived rejectful parental attitudes during childhood were positively correlated with problematic Internet use in adulthood, with emotional deprivation serving as a partial mediator in this relationship (Arifoglu and Artan, 2024). The relationship between parenting and child technology use appears to be mediated by factors such as basic psychological needs satisfaction and positive outcome expectations regarding technology use (Wang and Lei, 2022).

Based on the theoretical frameworks and empirical evidence reviewed above, this study proposes an integrated model examining the relationships between parenting styles, mobile phone addiction, and behavioral-emotional problems in children with ADHD. We hypothesize that: H1: Negative parenting styles (rejection and overprotection) will be positively associated with behavioral and emotional problems in children with ADHD, while positive parenting (emotional warmth) will be negatively associated with these problems. H2: Parenting styles will significantly influence mobile phone addiction levels, with negative parenting associated with higher addiction and positive parenting associated with lower addiction. H3: Mobile phone addiction will partially or fully mediate the relationship between parenting styles and behavioral-emotional problems in children with ADHD.

While acknowledging potential bidirectional links, the primary model posits mobile phone addiction as a mediator, consistent with theoretical frameworks suggesting that maladaptive digital engagement can be a proximal mechanism exacerbating psychological difficulties, particularly in children with ADHD who are vulnerable to dysregulated use. This mediation model extends previous research by examining mobile phone addiction as a potential mechanism linking parenting to child outcomes in the specific context of ADHD. Understanding these relationships has important implications for developing targeted interventions that address both family dynamics and technology use in this vulnerable population.

## 2 Research methods

### 2.1 Research participants and grouping

This cross-sectional study was conducted from November 2023 to June 2024 at three pediatric ADHD specialty clinics in urban settings. The study protocol was reviewed and approved by the Ethics Committee of Linhai Maternal and Child Health Hospital (Approval No.: [YJLFBY202417]; Approval Date: 30 December 2024). The study was conducted in accordance with the ethical standards of the Declaration of Helsinki. The study protocol was approved by the institutional review board, and all participants provided informed consent (parents) and assent (children aged 7 and above).

Participants included 232 children diagnosed with ADHD and their primary caregivers. Inclusion criteria were: (1) age between 6 and 17 years; (2) confirmed diagnosis of ADHD according to DSM-5 criteria by a qualified child psychiatrist or developmental pediatrician within the past 12 months; (3) no changes in ADHD medication regimen in the past 4 weeks if on medication; (4) regular access to a mobile phone (either personal or shared family device) for at least 6 months; (5) ability to read and understand questionnaires in the study language. Exclusion criteria included: (1) presence of autism spectrum disorder, intellectual disability (IQ < 70), or psychotic disorders; (2) significant sensory impairments (e.g., severe visual or hearing loss) that would substantially interfere with mobile phone use; (3) current participation in family therapy or parent training programs; (4) major medical conditions requiring intensive treatment (e.g., unstable severe cardiovascular, neurological, or autoimmune diseases).

The final sample consisted of 232 children (147 boys, 85 girls; mean age = 11.3 years, SD = 2.8) diagnosed with ADHD. Based on clinical assessment, 98 children (42.2%) met criteria for ADHD combined presentation, 89 (38.4%) for predominantly inattentive presentation, and 45 (19.4%) for predominantly hyperactive–impulsive presentation. Regarding medication status, 156 children (67.2%) were receiving pharmacological treatment for ADHD, while 76 (32.8%) were medication-naive or had discontinued medication. The sample was divided into three age groups for developmental comparisons: early childhood (6–9 years, *n* = 78), middle childhood (10–13 years, *n* = 92), and adolescence (14–17 years, *n* = 62). This grouping aligns with key developmental stages: 6–9 years (consolidation of self-regulation in primary school), 10–13 years (transition to adolescence with increasing peer influence), and 14–17 years (heightened autonomy seeking and identity formation).

### 2.2 Research instruments

#### 2.2.1 Child self-report measures

The study employed two primary self-report instruments for children. First, the Swanson, Nolan, and Pelham-IV (SNAP-IV) Rating Scale (26-item version) was utilized to assess ADHD symptom severity (Hall et al., 2020). This scale includes items corresponding to the 18 DSM-5 symptoms of ADHD (9 for inattention, 9 for hyperactivity-impulsivity) plus 8 items assessing oppositional defiant symptoms. Children rated each item on a 4-point scale (0 = not at all, 1 = just a little, 2 = quite a bit, 3 = very much). The scale demonstrated good internal consistency in our sample (Cronbach’s α = 0.89 for inattention, 0.87 for hyperactivity-impulsivity).

Second, the Mobile Phone Use Dependence Self-Assessment Questionnaire (13-item version) was administered to evaluate problematic mobile phone use (Arrindell et al., 1999). This instrument assesses core addiction components including: (1) loss of control over usage; (2) withdrawal symptoms when unable to use the phone; (3) tolerance (needing increasing amounts of use); (4) negative consequences on daily functioning; (5) preoccupation with phone use. Items are rated on a 5-point Likert scale (1 = strongly disagree to 5 = strongly agree), with higher scores indicating greater addiction severity. The scale showed excellent reliability (α = 0.91) and has been validated for use with children as young as 8 years. For the SNAP-IV, mean scores ≥1.78 for inattention items and ≥1.44 for hyperactivity-impulsivity items indicate clinically significant symptoms. For the Mobile Phone Use Dependence Self-Assessment Questionnaire, total scores range from 13 to 65, with scores >39 indicating moderate addiction and >52 indicating severe addiction.

#### 2.2.2 Parent-rated assessments

Parents completed three comprehensive assessment tools. The parent version of the SNAP-IV provided an independent assessment of child ADHD symptoms, using the same 26-item format as the child version. Inter-rater reliability between parent and child ratings was moderate (ICC = 0.68), which is typical for parent–child agreement on behavioral symptoms. Therefore, in line with established practice that prioritizes parent report as the more reliable and contextually broad assessment for externalizing behaviors, the parent-rated SNAP-IV scores were used as the primary measure for all subsequent statistical analyses.

The Child Behavior Checklist Brief Form (CBCL/6-18) was used to assess the study’s core outcome, children’s emotional-behavioral problems (Achenbach and Rescorla, 2001). This construct was operationalized using the instrument‘s three primary broadband indices: the Total Problems T-score (a global index of overall dysfunction), the Internalizing Problems T-score (combining anxious/depressed, withdrawn/depressed, and somatic complaints), and the Externalizing Problems T-score (combining rule-breaking and aggressive behavior). Parents rate each item as 0 (not true), 1 (somewhat or sometimes true), or 2 (very true or often true) based on the child’s behavior over the past 6 months. The CBCL demonstrated strong psychometric properties (α = 0.82–0.94 for syndrome scales). In the statistical analyses, these three T-scores (Total, Internalizing, and Externalizing) were utilized to comprehensively capture the multidimensional nature of emotional-behavioral problems.

The Egna Minnen Beträffande Uppfostran (EMBU) Parenting Style Scale was employed to assess parenting practices (Li et al., 2012). The Chinese version used in this study consists of 66 items measuring parenting across six paternal dimensions and five maternal dimensions. Key dimensions include: (1) emotional warmth and understanding (assessing affection, support, and acceptance); (2) punishment and strictness (harsh discipline and criticism); (3) over-interference and overprotection (excessive control and intrusion); (4) rejection and denial (hostility and neglect); (5) favoritism (differential treatment among siblings). Items are rated on a 4-point scale (1 = never, 2 = sometimes, 3 = often, 4 = always). The EMBU showed good reliability across subscales (α = 0.73–0.89).

#### 2.2.3 Demographic and clinical information

A structured demographic questionnaire collected information on child age, gender, grade level, ADHD subtype, medication status, age at diagnosis, comorbid conditions, family structure, parental education, occupation, and household income. Additional items assessed mobile phone access patterns, including age of first phone use, daily usage time, primary phone activities, and parental monitoring of phone use.

### 2.3 Measurement procedures and quality control

Data collection followed a standardized protocol to ensure consistency and quality. The research team consisted of trained graduate students in clinical psychology and experienced research nurses who underwent a two-day training workshop covering study procedures, ethical considerations, and standardized administration of all instruments. Assessment sessions were conducted in quiet clinic rooms during regular clinic hours. To minimize fatigue effects, the protocol was divided into two parts: (1) child assessments completed first with a research assistant present to provide clarification if needed (approximately 30–45 min); (2) parent assessments completed independently while children engaged in a break activity (approximately 45–60 min). For younger children (ages 6–8), questionnaires were administered in interview format with visual aids to ensure comprehension. Quality control measures included: (1) double data entry with discrepancy resolution; (2) range checks to identify out-of-range values; (3) consistency checks for reverse-coded items; (4) missing data assessment with follow-up for questionnaires with >10% missing items; (5) random auditing of 10% of questionnaires for accuracy; (6) weekly team meetings to address procedural questions and maintain standardization.

### 2.4 Statistical analysis plan

We anticipated detecting a moderate effect size (*f*^2^ = 0.2). Using G*Power software with α = 0.05 and power (1-β) = 0.80, the calculated minimum sample size was 191 participants. The final sample size of 232 participants met this requirement.

Statistical analyses were performed using SPSS version 27.0 and the PROCESS macro version 4.0 (Hayes, 2022). Parent-reported measures were used for parenting styles (EMBU) and child emotional-behavioral problems (CBCL). The child self-reported measure was used for mobile phone addiction.

The analysis plan proceeded in several stages to comprehensively examine the research hypotheses. Preliminary analyses included descriptive statistics (means, standard deviations, ranges) for all study variables and assessment of distributional properties. Variables with absolute skewness > 3 or absolute kurtosis > 10 were transformed using logarithmic transformation for positively skewed variables and square root transformation for moderately skewed variables (Kline, 2015; Field, 2018). The skewness and kurtosis of all key continuous variables were examined (see Supplementary Table S1 for values). As all absolute skewness values were <1 and all absolute kurtosis values were <1.1, no variables required transformation for normality. Missing data patterns were examined, and Little’s MCAR test was conducted to assess whether data were missing completely at random. For variables with <5% missing data, pairwise deletion was used; for variables with 5–20% missing data, multiple imputation was employed using the expectation–maximization algorithm.

Group comparisons were conducted using independent samples *t*-tests for comparisons between two groups (e.g., gender, medication status) and one-way ANOVA for comparisons among three or more groups, with *p* < 0.05 considered statistically significant (e.g., age groups, ADHD subtypes). Prior to these analyses, the underlying assumptions were verified. The homogeneity of variances was assessed using Levene’s test, and the normality of residuals was examined using the Shapiro–Wilk test. For all reported t-tests and ANOVAs, the assumptions of homogeneity of variance were met (all *p*-values for Levene’s test > 0.05). Effect sizes were calculated using Cohen’s d for *t*-tests and partial eta-squared for ANOVAs. Post-hoc comparisons used Bonferroni correction to control for Type I error.

Correlation analyses examined bivariate associations among parenting styles, mobile phone addiction, and behavioral-emotional problems. Both Pearson correlations (for normally distributed continuous variables) and Spearman correlations (for ordinal or non-normal variables) were calculated. Partial correlations controlling for age, gender, and medication status were also computed to assess relationships independent of these potential confounds.

The primary mediation analyses were conducted using Hayes’ PROCESS macro (Model 4 for simple mediation) with 5,000 bootstrap samples to generate bias-corrected 95% confidence intervals for indirect effects (Preacher and Hayes, 2008). Separate models were tested for: (1) negative parenting (rejection and overprotection combined) → mobile phone addiction → behavioral-emotional problems; (2) positive parenting (emotional warmth) → mobile phone addiction → behavioral-emotional problems. Covariates included in all models were child age, gender, ADHD subtype, medication status, family income, and parental education level. For each mediation model, we examined: (1) the total effect (c path) of parenting on child outcomes; (2) the direct effect (c’ path) of parenting on outcomes controlling for the mediator; (3) the indirect effect (a × b path) through mobile phone addiction; (4) the proportion of the total effect mediated. Mediation was considered significant if the 95% confidence interval for the indirect effect did not include zero.

Sensitivity analyses included: (1) testing models separately for maternal and paternal parenting; (2) examining moderated mediation with age group as a moderator; (3) testing alternative models with behavioral problems as mediator between parenting and phone addiction.

## 3 Results

### 3.1 Sample characteristics

The demographic and clinical characteristics of the sample are presented in Table 1. The mean age of participants was 11.3 ± 2.8 years, with a male predominance (147, 63.4%) consistent with ADHD prevalence patterns. One hundred and fifty-six of children (67.2%) were receiving medication for ADHD, with stimulants being the most common treatment (54.3%). Specifically, 99 children (42.7%) met criteria for oppositional defiant disorder, 67 (28.9%) for anxiety disorders, and 46 (19.8%) for learning disabilities.

**Table 1:** Demographic and clinical characteristics of the sample (*N* = 232).

Variable	*n* (%) or Mean ± SD
Age (years)	11.3 ± 2.8
Gender	
Male	147 (63.4%)
Female	85 (36.6%)
ADHD presentation	
Combined	98 (42.2%)
Inattentive	89 (38.4%)
Hyperactive–Impulsive	45 (19.4%)
Medication status	
Currently medicated	156 (67.2%)
Not medicated	76 (32.8%)
Age at ADHD diagnosis	7.8 ± 2.1
Comorbid conditions	
Oppositional defiant disorder	99 (42.7%)
Anxiety disorders	67 (28.9%)
Learning disabilities	46 (19.8%)
Mood disorders	31 (13.4%)
Mobile phone access	
Own phone	168 (72.4%)
Shared family phone	64 (27.6%)
Daily phone use (hours)	3.2 ± 1.8
Family structure	
Two-parent household	178 (76.7%)
Single-parent household	42 (18.1%)
Other arrangements	12 (5.2%)
Maternal education	
High school or below	89 (38.4%)
College/university	118 (50.9%)
Graduate degree	25 (10.8%)
Annual household income	
<$30,000	56 (24.1%)
$30,000–60,000	98 (42.2%)
>$60,000	78 (33.6%)

### 3.2 Scale scores and group comparisons

**Figure 1:**
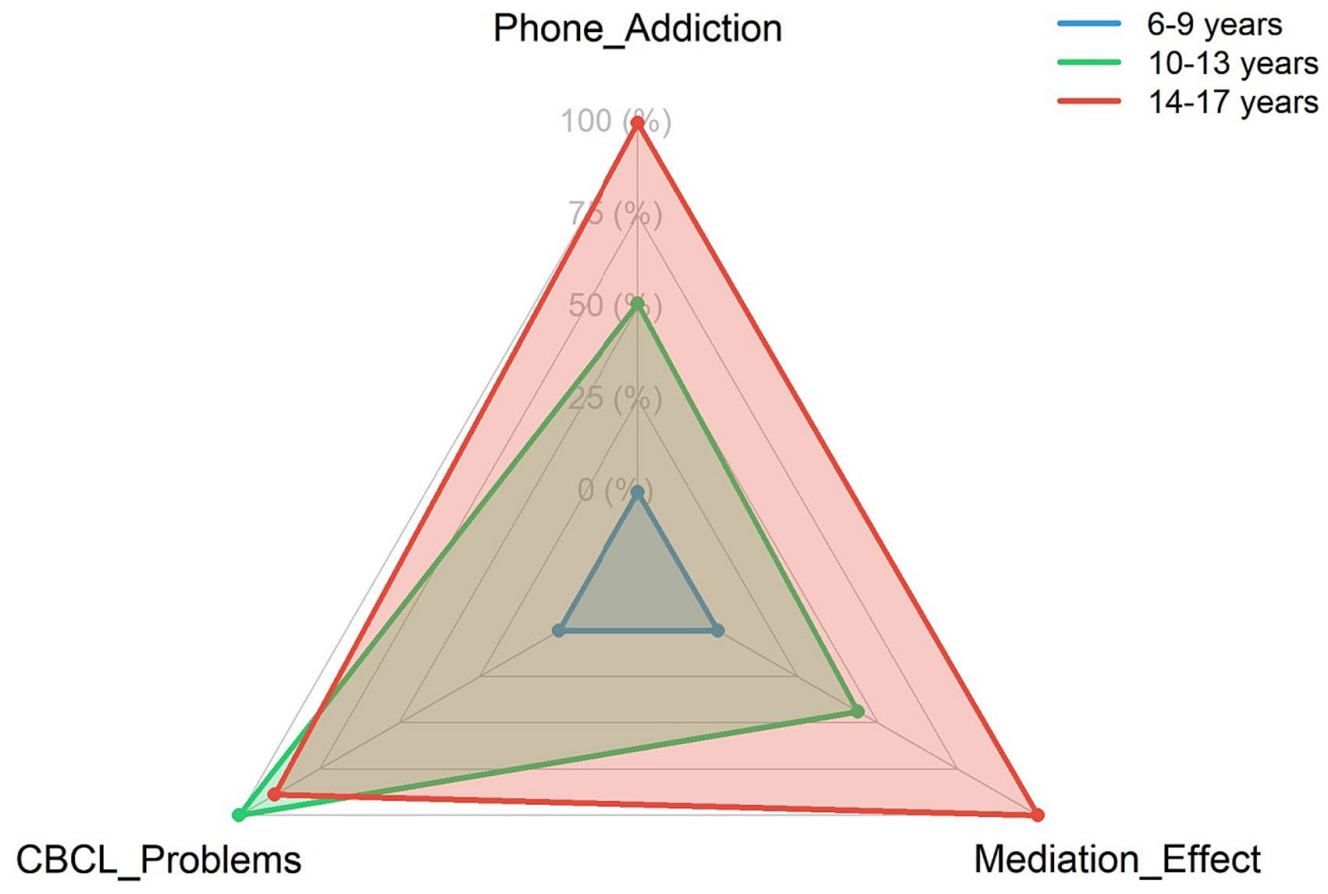
Age group differences in mobile phone addiction and behavioral problems.

**Figure 2:**
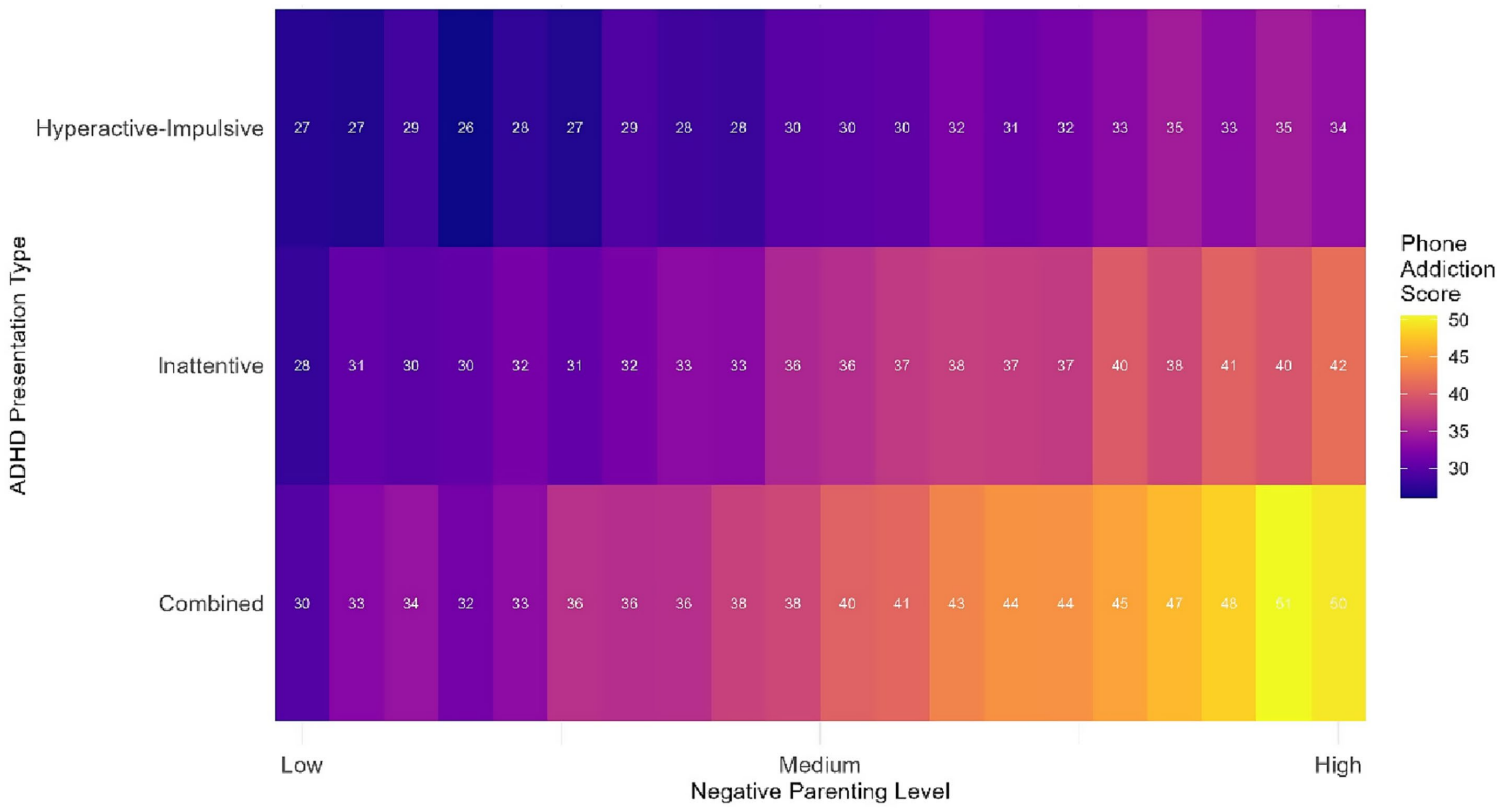
Interaction between parenting styles and ADHD presentation on mobile phone addiction. Negative parenting levels were categorized as: Low (<1 SD below mean), Moderate (within ±1 SD of mean), High (>1 SD above mean).

Table 2 presents descriptive statistics for all primary study variables and comparisons across key demographic groups. Significant age group differences emerged for mobile phone addiction, with adolescents showing the highest scores (38.1 ± 11.2, see Figure 1). Gender differences were observed for externalizing problems, with boys (67.3 ± 10.1) exhibiting higher scores than girls (62.9 ± 9.8). Children with the combined ADHD presentation showed significantly higher scores on both SNAP-IV subscales and CBCL externalizing problems compared to other presentations (*p* < 0.001), and as illustrated in Figure 2, they also demonstrated the strongest association between negative parenting and mobile phone addiction.

**Table 2:** Descriptive statistics and group comparisons for primary variables.

Variable	Total sample	Age groups			Gender	
	Mean ± SD	6–9 years (a)	10–13 years (b)	14–17 years (c)	Male	Female
Child-rated measures						
SNAP-IV inattention	18.3 ± 4.8	17.6 ± 4.9^c^	18.2 ± 4.6	19.3 ± 4.8^a^	18.7 ± 5.3	17.6 ± 5.0
SNAP-IV hyperactivity	15.3 ± 5.3	14.6 ± 5.2	15.1 ± 5.6	16.4 ± 4.9	16.4 ± 5.7*	14.2 ± 5.6
Mobile phone addiction	32.5 ± 11.1	27.5 ± 9.4^bc^	32.8 ± 10.6^ac^	38.1 ± 11.2^ab^	33.1 ± 11.5	31.2 ± 10.9
Parent-rated measures						
CBCL internalizing	62.3 ± 9.8	61.7 ± 9.9	63.1 ± 9.5	61.9 ± 10.1	61.2 ± 9.5	64.2 ± 10.0*
CBCL externalizing	65.7 ± 10.1	63.8 ± 9.8^b^	67.1 ± 10.6^a^	66 ± 9.6	67.3 ± 10.1**	62.9 ± 9.8
CBCL total problems	64.5 ± 9.3	63.3 ± 9.5	65.9 ± 9.1	64.1 ± 9.4	65.2 ± 9.6	64.1 ± 9.3
Parenting styles						
Maternal warmth	42.4 ± 8.2	43 ± 8.3	42.1 ± 7.7	42.3 ± 8.9	41.9 ± 8.8	43.0 ± 8.5
Maternal rejection	18.3 ± 5.5	18.5 ± 5.8	18 ± 5.5	18.6 ± 5.2	18.9 ± 5.4	18.1 ± 5.1
Maternal overprotection	28.6 ± 6	28.6 ± 6.4	28.2 ± 5.9	29.2 ± 5.7	29.1 ± 6.3	28.5 ± 6.0
Paternal warmth	40 ± 8.5	40.8 ± 7.7	40.6 ± 8.4	38.1 ± 9.3	39.3 ± 9.2	40.4 ± 8.9
Paternal rejection	16.8 ± 5.1	16.4 ± 5	16.8 ± 5.5	17.4 ± 4.5	17.5 ± 5.1	16.7 ± 4.8
Paternal overprotection	26.3 ± 5.6	26.4 ± 5.6	25.8 ± 5.7	26.9 ± 5.6	26.6 ± 5.9	26.0 ± 5.6

Age groups: Different superscript letters (a, b, c) within a row indicate statistically significant differences between age groups based on one-way ANOVA with Bonferroni *post-hoc* test (*P* < 0.05).

Gender: An asterisk next to the mean indicates a significant gender difference based on independent samples *t*-test (*P < 0.05, **P < 0.01, ***P < 0.001).

CBCL, Child Behavior Checklist; SNAP-IV, Swanson, Nolan, and Pelham Rating Scale, Version IV.

### 3.3 Correlation matrix

**Figure 3:**
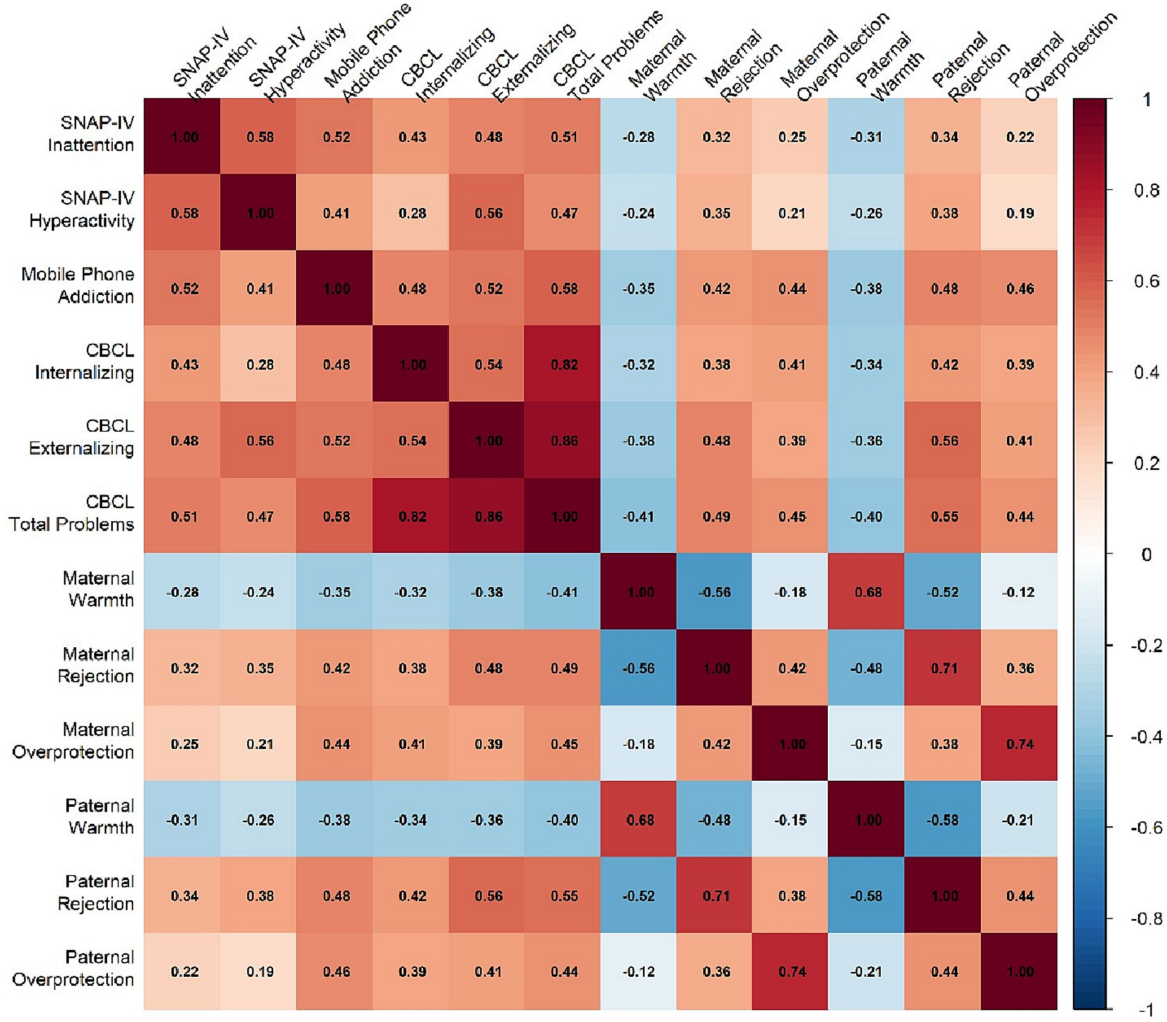
Correlation heatmap of study variables.

Bivariate correlations among primary study variables are presented in Table 3 and visualized in Figure 3. As hypothesized, negative parenting dimensions (rejection and overprotection) showed significant positive correlations with mobile phone addiction (*r* = 0.42–0.48, *p* < 0.001) and behavioral-emotional problems (*r* = 0.38–0.56, *p* < 0.001). Conversely, parental emotional warmth was negatively correlated with both phone addiction (*r* = −0.35 to −0.38, *p* < 0.001) and child psychopathology (*r* = −0.32 to −0.41, *p* < 0.001). Mobile phone addiction showed strong positive correlations with all CBCL problem scales (*r* = 0.45–0.58, *p* < 0.001). The correlation heatmap (Figure 2) clearly illustrates the clustering of negative parenting behaviors and their consistent associations with adverse child outcomes.

**Table 3:** Correlation matrix of study variables.

Variable	1	2	3	4	5	6	7	8	9	10	11
1. SNAP-IV inattention	–										
2. SNAP-IV hyperactivity	0.58***	–									
3. Mobile phone addiction	0.52***	0.41***	–								
4. CBCL internalizing	0.43***	0.28***	0.48***	–							
5. CBCL externalizing	0.48***	0.56***	0.52***	0.54***	–						
6. CBCL total problems	0.51***	0.47***	0.58***	0.82***	0.86***	–					
7. Maternal warmth	−0.28***	−0.24***	−0.35***	−0.32***	−0.38***	−0.41***	–				
8. Maternal rejection	0.32***	0.35***	0.42***	0.38***	0.48***	0.49***	−0.56***	–			
9. Maternal overprotection	0.25***	0.21**	0.44***	0.41***	0.39***	0.45***	−0.18**	0.42***	–		
10. Paternal warmth	−0.31***	−0.26***	−0.38***	−0.34***	−0.36***	−0.40***	0.68***	−0.48***	−0.15*	–	
11. Paternal rejection	0.34***	0.38***	0.48***	0.42***	0.56***	0.55***	−0.52***	0.71***	0.38***	−0.58***	–
12. Paternal overprotection	0.22**	0.19**	0.46***	0.39***	0.41***	0.44***	−0.12	0.36***	0.74***	−0.21**	0.44***

*p < 0.05, **p < 0.01, ***p < 0.001.

### 3.4 Mediation model testing

**Figure 4:**
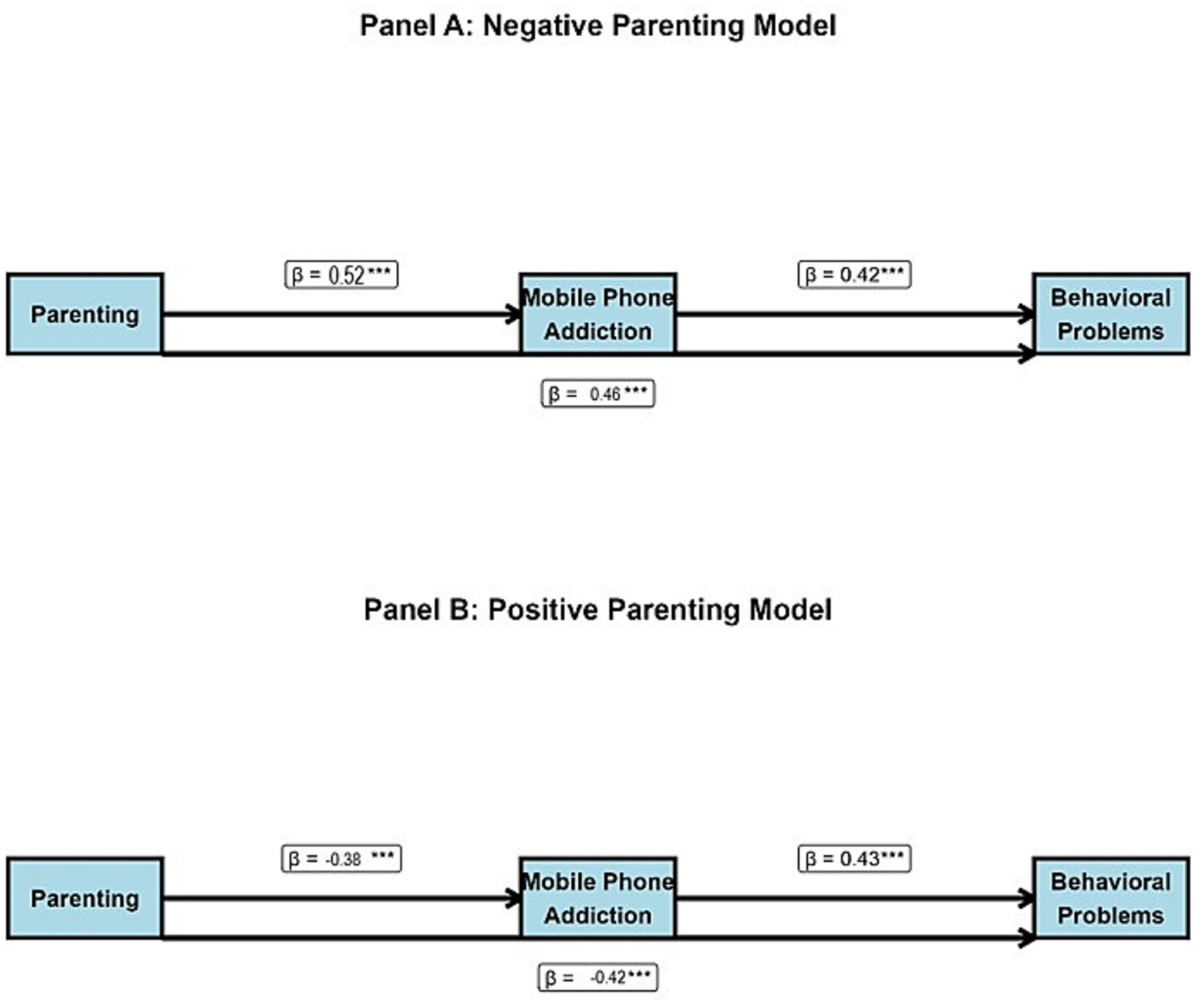
Mediation models with standardized path coefficients. *** value represent statistically significant.

The primary mediation analyses examined whether mobile phone addiction mediated the relationship between parenting styles and behavioral-emotional problems. Results of the PROCESS analysis are presented in Table 4 and illustrated in Figure 4. The mediation analysis revealed significant indirect effects for both negative and positive parenting models. For negative parenting, the total effect on behavioral problems was significant (β = 0.68, 95% CI: 0.50–0.86), with mobile phone addiction mediating 32.4% of this effect. The indirect path through phone addiction was statistically significant (β = 0.22, 95% CI: 0.14–0.31).

**Table 4:** Mediation analysis results: mobile phone addiction as mediator.

Path/effect	*B*	SE	*t*	*p*	95% CI
Model 1: Negative parenting → Phone addiction → Total problems					
Total effect (c)	0.68	0.09	7.56	<0.001	[0.50, 0.86]
Direct effect (c’)	0.46	0.08	5.75	<0.001	[0.30, 0.62]
a path (Parenting → Phone addiction)	0.52	0.07	7.43	<0.001	[0.38, 0.66]
b path (Phone addiction → Problems)	0.42	0.06	7.00	<0.001	[0.30, 0.54]
Indirect effect (a × b)	0.22	0.04			[0.14, 0.31]
Proportion mediated	32.4%				
Model 2: Positive parenting → Phone addiction → Total problems					
Total effect (c)	−0.58	0.08	−7.25	<0.001	[−0.74, −0.42]
Direct effect (c’)	−0.42	0.08	−5.25	<0.001	[−0.58, −0.26]
a path (Parenting → Phone addiction)	−0.38	0.06	−6.33	<0.001	[−0.50, −0.26]
b path (Phone Addiction → problems)	0.43	0.06	7.17	<0.001	[0.31, 0.55]
Indirect effect (a × b)	−0.16	0.04			[−0.24, −0.09]
Proportion mediated	27.6%				

All models controlled for child age, gender, ADHD subtype, medication status, family income, and parental education. Bootstrap samples = 5,000. CI, confidence interval.

**Figure 5:**
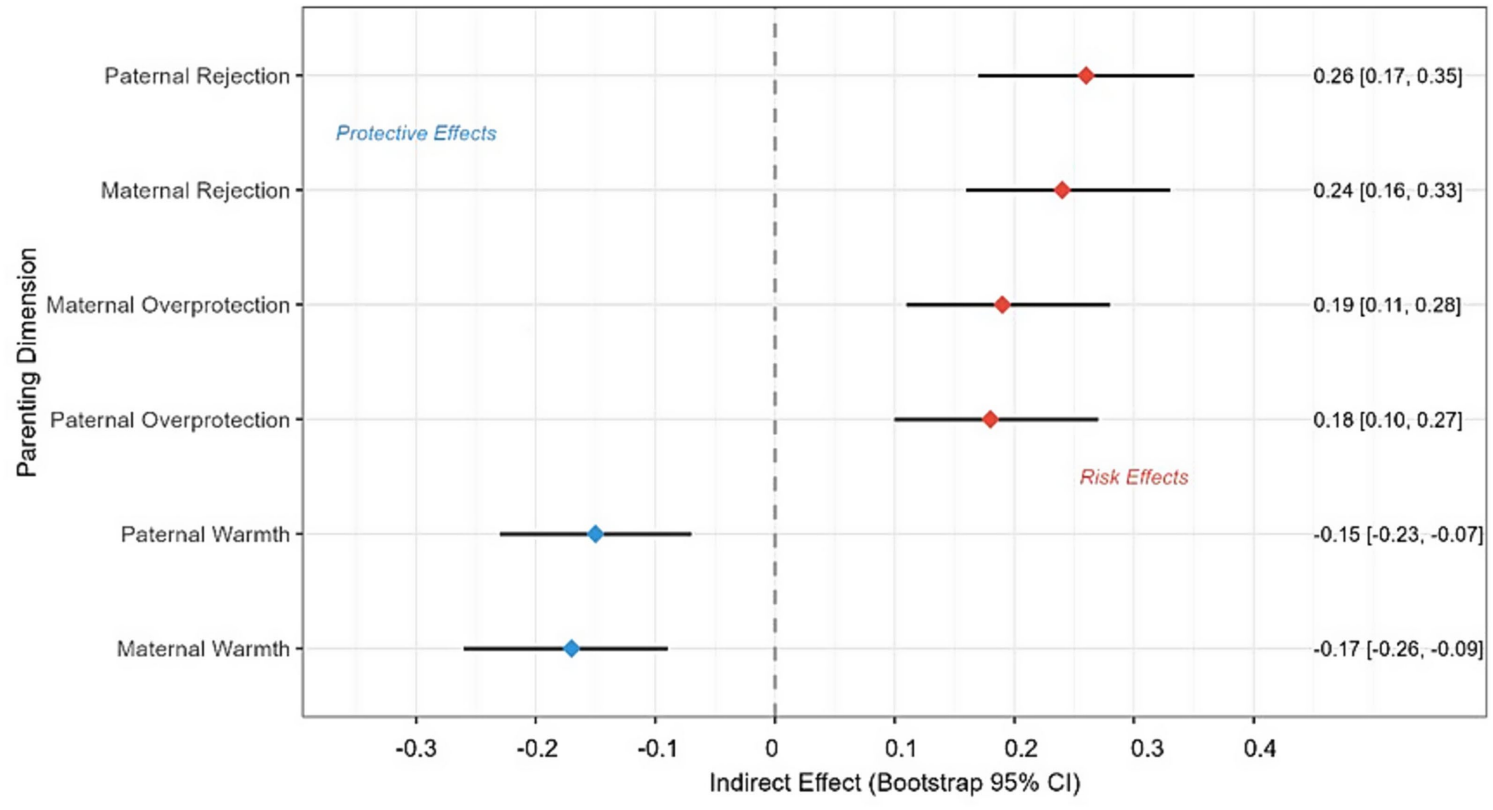
Bootstrap confidence intervals for indirect effects across different parenting dimensions.

As shown in Figure 5, bootstrap confidence intervals confirmed significant indirect effects for all parenting dimensions examined, with Paternal Rejection showing the strongest indirect effect through mobile phone addiction. Figure 5 presents a forest plot showing bootstrap confidence intervals for indirect effects of different parenting dimensions on behavioral-emotional problems through mobile phone addiction. The plot displays six horizontal lines with confidence intervals: Maternal Rejection (indirect effect = 0.24, 95% CI: 0.16–0.33), Paternal Rejection (indirect effect = 0.26, 95% CI: 0.17–0.35), Maternal Overprotection (indirect effect = 0.19, 95% CI: 0.11–0.28), Paternal Overprotection (indirect effect = 0.18, 95% CI: 0.10–0.27), Maternal Warmth (indirect effect = −0.17, 95% CI: −0.26 to −0.09), and Paternal Warmth (indirect effect = −0.15, 95% CI: −0.23 to −0.07).

### 3.5 Sensitivity analysis

The sensitivity analysis did not fundamentally change the results of the main analysis (Supplementary Tables S2–S4).

## 4 Discussion

This study examined the complex relationships between parenting styles, mobile phone addiction, and behavioral-emotional problems in children with ADHD, revealing mobile phone addiction as a significant mediating mechanism. Our findings provide novel insights into how contemporary digital behaviors may serve as a pathway through which family dynamics influence child psychopathology in this vulnerable population.

The results strongly support our hypothesized model, demonstrating that parenting styles exert both direct and indirect effects on behavioral-emotional problems in children with ADHD. The finding that negative parenting practices (rejection and overprotection) were associated with increased psychopathology aligns with extensive literature documenting the detrimental effects of harsh, critical, and overly controlling parenting on child mental health (Cueli et al., 2024; Deault, 2010). The magnitude of these associations in our ADHD sample (*r* = 0.41–0.56) exceeded those typically reported in general population studies (*r* = 0.25–0.35), suggesting heightened vulnerability. This suggests that children with ADHD may be especially vulnerable to the effects of negative parenting, possibly due to their already compromised self-regulation capacities and heightened emotional reactivity.

The protective effect of parental emotional warmth on child outcomes is consistent with attachment theory and research demonstrating that supportive, accepting parenting fosters resilience and adaptive functioning (Gao et al., 2024). In the context of ADHD, where children face numerous daily challenges and frustrations, parental warmth may be particularly critical for maintaining self-esteem and motivation. Our findings suggest that even in the presence of significant neurodevelopmental challenges, positive parenting can serve as a buffer against the development of comorbid psychopathology.

Perhaps most importantly, our study identifies mobile phone addiction as a significant mediator linking parenting styles to child outcomes. The finding that approximately 30% of the parenting effect operates through phone addiction highlights the importance of considering digital behaviors in understanding family influences on child psychopathology. This mediation effect can be understood through multiple theoretical lenses. From a social learning perspective, children may model parental technology use patterns and emotional regulation strategies (Zhang, 2025). Parents who are rejecting or overcontrolling may inadvertently push children toward digital devices as a source of comfort and autonomy (Zhu et al., 2022). Conversely, emotionally warm parents may foster secure attachment relationships that reduce the need for excessive digital engagement as a coping mechanism (Detnakarintra et al., 2020).

The stronger mediation effects observed in adolescents compared to younger children (shown in Figure 3) warrant particular attention. This developmental pattern likely reflects the increasing importance of peer relationships and autonomy during adolescence, combined with greater unsupervised access to technology. For adolescents with ADHD, who often struggle with peer relationships and academic demands, mobile phones may offer an particularly appealing escape from real-world challenges (Yum et al., 2025). The instant gratification and constant stimulation provided by smartphones may be especially reinforcing for individuals with ADHD-related reward processing differences (Deng et al., 2021). Furthermore, the interaction between ADHD presentation and parenting styles shown in Figure 2 suggests that children with combined-type ADHD may be particularly vulnerable to developing mobile phone addiction in the context of negative parenting.

Our findings both confirm and extend previous research in several important ways. The association between parenting styles and ADHD symptom severity replicates numerous studies demonstrating that family factors influence the expression and course of ADHD (Samimy, 2022; Paiva et al., 2024). However, our study goes beyond symptom severity to examine broader patterns of comorbidity, revealing that parenting styles are associated with the full spectrum of internalizing and externalizing problems commonly seen in ADHD.

The rates of mobile phone addiction in our sample (with mean scores indicating moderate to high levels of problematic use) are consistent with recent studies suggesting that youth with ADHD are at elevated risk for technology addiction (Arrindell et al., 1999; Rohner and Lansford, 2017). The correlation between ADHD symptoms and phone addiction (*r* = 0.52 for inattention, *r* = 0.41 for hyperactivity) aligns closely with a recent meta-analysis reporting pooled correlations in the 0.40–0.50 range (Zhang et al., 2021). Our study extends this literature by demonstrating that phone addiction is not merely correlated with ADHD symptoms but serves as a mechanism linking environmental factors (parenting) to clinical outcomes.

Compared to studies of parenting and technology use in typically developing youth (Zhang et al., 2025; Wang and Lei, 2022), our ADHD sample showed stronger associations between negative parenting and phone addiction. This suggests that the combination of ADHD-related vulnerabilities and suboptimal parenting may create a “perfect storm” for the development of problematic technology use. The partial mediation effects we observed are consistent with recent studies examining digital behaviors as mediators of family influences, though most previous research has focused on general population samples rather than clinical populations (Liu et al., 2021; Elsaesser et al., 2017).

Our findings have important theoretical implications for understanding ADHD-related impairment in the digital age. The identification of mobile phone addiction as a mediating mechanism suggests that contemporary models need to incorporate digital behaviors as a key domain of functioning, moving beyond traditional focus on academic, social, and family impairment. From a clinical perspective, our results indicate that interventions for children with ADHD should address both parenting practices and digital device management simultaneously. Current evidence-based treatments, including behavioral parent training and family therapy, would benefit from incorporating modules on digital parenting practices, such as setting appropriate limits and modeling healthy technology use. The partial mediation effects observed suggest that improving parenting alone may not be sufficient; concurrent intervention targeting phone addiction through cognitive-behavioral strategies and skills training is necessary. Given the stronger effects in adolescents, developmentally tailored interventions acknowledging peer relationships and autonomy needs are particularly important. For prevention, early identification of at-risk families with negative parenting patterns could enable targeted intervention before problematic phone use becomes entrenched.

Several limitations should be acknowledged. The cross-sectional design precludes causal inferences, necessitating longitudinal research to establish temporal precedence and examine developmental trajectories. Our reliance on self-report and parent-report measures may have introduced bias, suggesting future research should incorporate objective measures of phone use, observational assessments of parent–child interactions, and multi-informant ratings. The sample from clinical settings with high medication rates (67%) may limit generalizability, particularly to children with milder ADHD symptoms or diverse cultural contexts. Additionally, we did not assess specific types of phone use (social media, gaming, communication), which may have differential relationships with psychopathology. Future studies should examine not just quantity but quality of phone use, including content consumed and social interactions facilitated. While we controlled for several confounds, unmeasured variables such as parental psychopathology, parental phone use, peer relationships, and school functioning may influence the observed relationships. Future research should employ more comprehensive ecological assessments and prospective designs to better understand how parenting, technology use, and child psychopathology influence each other reciprocally over time. Fourth, the emotional-behavioral problems outcome relied on parent-report questionnaires (the CBCL) rather than semi-structured diagnostic interviews. While these questionnaires are well-validated for dimensional assessment, they do not provide formal clinical diagnoses. Additionally, the emotional-behavioral problems outcome was measured using the CBCL, a broadband screening instrument. While its Total, Internalizing, and Externalizing scores provide valuable composite indices, they are not equivalent to diagnoses from disorder-specific clinical scales.

## 5 Conclusion

This study provides compelling evidence that mobile phone addiction serves as a significant mediator in the relationship between parenting styles and behavioral-emotional problems in children with ADHD. The findings highlight the need for integrated interventions that address both family dynamics and digital behaviors in this vulnerable population. As technology continues to play an increasingly central role in children’s lives, understanding how it intersects with neurodevelopmental conditions and family factors becomes critical for optimizing outcomes. Future longitudinal research should examine how these relationships evolve across development and evaluate targeted interventions that address the complex interplay between parenting, technology use, and child psychopathology in ADHD.

## Data Availability

The original contributions presented in the study are included in the article/Supplementary material, further inquiries can be directed to the corresponding author.
