# Supplementary material for: The impact of family parenting styles on behavioral and emotional problems in children with attention-deficit/hyperactivity disorder: the mediating role of mobile phone addiction
Source: Front Psychol. 2026 Mar 4;17:1729300. doi: 10.3389/fpsyg.2026.1729300 (PMC12996205; doi:10.3389/fpsyg.2026.1729300)
Supplement: Supplementary file 1 [file Table_1.DOC]

Table S1. Normality Indices (Skewness and Kurtosis) for Key Study Variables.

| Variable | N | Skewness | Std. Error | Kurtosis | Std. Error |
| --- | --- | --- | --- | --- | --- |
| Age | 232 | 0.110 | 0.160 | -1.039 | 0.318 |
| Age at Diagnosis | 232 | 0.244 | 0.160 | -0.454 | 0.318 |
| Daily Phone Use (Hours) | 232 | 0.173 | 0.160 | -0.272 | 0.318 |
| SNAP-IV_Inattention | 232 | -0.177 | 0.160 | -0.272 | 0.318 |
| SNAP-IV_Hyperactivity | 232 | -0.153 | 0.160 | 0.121 | 0.318 |
| Mobile Phone Addiction | 232 | 0.320 | 0.160 | -0.384 | 0.318 |
| CBCL_Internalizing Problems | 232 | 0.062 | 0.160 | -0.324 | 0.318 |
| CBCL_Externalizing Problems | 232 | -0.069 | 0.160 | -0.197 | 0.318 |
| CBCL_Total Problems | 232 | -0.150 | 0.160 | -0.276 | 0.318 |
| Maternal Warmth | 232 | -0.104 | 0.160 | -0.159 | 0.318 |
| Maternal Rejection | 232 | -0.064 | 0.160 | -0.339 | 0.318 |
| Maternal Overprotection | 232 | -0.096 | 0.160 | -0.050 | 0.318 |
| Paternal Warmth | 232 | 0.086 | 0.160 | 0.142 | 0.318 |
| Paternal Rejection | 232 | -0.090 | 0.160 | -0.265 | 0.318 |
| Paternal Overprotection | 232 | -0.105 | 0.160 | 0.243 | 0.318 |

*Note.* CBCL = Child Behavior Checklist; SNAP-IV = Swanson, Nolan, and Pelham Rating Scale, Version IV.

**Table S2. Sensitivity Analysis: Comparing Mediation Models for Maternal and Paternal Parenting Styles Separately.**

| **Path/Effect** | **B** | **SE** | **t** | ***P*** | **95% CI** |
| --- | --- | --- | --- | --- | --- |
| **Paternal Parenting Styles** |  |  |  |  |  |
| **Model 1:** Negative Parenting → Phone Addiction → Total Problems |  |  |  |  |  |
| Total effect (c) | 0.62 | 0.06 | 9.51 | 0.00 | [0.49, 0.74] |
| Direct effect (c') | 0.41 | 0.07 | 5.96 | 0.00 | [0.27, 0.54] |
| a path (Parenting → Phone Addiction) | 0.59 | 0.08 | 7.87 | 0.00 | [0.44, 0.74] |
| b path (Phone Addiction → Problems) | 0.35 | 0.06 | 6.19 | 0.00 | [0.24, 0.47] |
| Indirect effect (a × b) | 0.21 | 0.04 |  |  | [0.14, 0.29] |
| Proportion mediated | 33.9% |  |  |  |  |
| **Model 2:** Positive Parenting → Phone Addiction → Total Problems |  |  |  |  |  |
| Total effect (c) | -0.51 | 0.08 | -6.73 | 0.00 | [-0.66, -0.36] |
| Direct effect (c') | -0.36 | 0.07 | -5.34 | 0.00 | [-0.49, -0.23] |
| a path (Parenting → Phone Addiction) | -0.34 | 0.09 | -3.80 | 0.00 | [-0.51, -0.16] |
| b path (Phone Addiction → Problems) | 0.45 | 0.05 | 8.57 | 0.00 | [0.35, 0.55] |
| Indirect effect (a × b) | -0.15 | 0.04 |  |  | [-0.24, -0.07] |
| Proportion mediated | 29.4% |  |  |  |  |
| **Maternal Parenting Styles** |  |  |  |  |  |
| **Model 1**: Negative Parenting → Phone Addiction → Total Problems |  |  |  |  |  |
| Total effect (c) | 0.52 | 0.06 | 8.24 | 0.00 | [0.40, 0.65] |
| Direct effect (c') | 0.31 | 0.07 | 4.58 | 0.00 | [0.17, 0.44] |
| a path (Parenting → Phone Addiction) | 0.56 | 0.07 | 8.04 | 0.00 | [0.43, 0.70] |
| b path (Phone Addiction → Problems) | 0.39 | 0.06 | 6.50 | 0.00 | [0.27, 0.50] |
| Indirect effect (a × b) | 0.22 | 0.04 |  |  | [0.15, 0.29] |
| Proportion mediated | 42.3% |  |  |  |  |
| **Model 2:** Positive Parenting → Phone Addiction → Total Problems |  |  |  |  |  |
| Total effect (c) | -0.55 | 0.07 | -7.35 | 0.00 | [-0.69, -0.40] |
| Direct effect (c') | -0.37 | 0.07 | -5.42 | 0.00 | [-0.50, -0.23] |
| a path (Parenting → Phone Addiction) | -0.41 | 0.09 | -4.74 | 0.00 | [-0.59, -0.24] |
| b path (Phone Addiction → Problems) | 0.43 | 0.05 | 8.06 | 0.00 | [0.32, 0.53] |
| Indirect effect (a × b) | -0.18 | 0.04 |  |  | [-0.26, -0.10] |
| Proportion mediated | 32.7% |  |  |  |  |

Note: All models controlled for child age, gender, ADHD subtype, medication status, family income, and parental education. Bootstrap samples = 5,000. CI = confidence interval.

**Table S3. Sensitivity Analysis: Moderated Mediation Models with Age Group as a Moderator.**

| **Path/Effect** | **B** | **SE** | **t** | ***P*** | **95% CI** | **Proportion mediated** |
| --- | --- | --- | --- | --- | --- | --- |
| **Model 1**: Negative Parenting → Phone Addiction → Total Problems |  |  |  |  |  |  |
| Total effect (c) | 0.32 | 0.03 | 9.77 | 0.00 | [0.26, 0.39] |  |
| Direct effect (c') | 0.21 | 0.04 | 5.79 | 0.00 | [0.14, 0.28] | 65.6% |
| Indirect effect (a × b) | 0.11 | 0.02 |  |  | [0.07, 0.16] | 34.4% |
| Ind 1 (Parenting → Phone Addiction→ Problems) | 0.11 | 0.02 |  |  | [0.08, 0.16] | 100% |
| Ind 2 (Parenting →Age → Problems) | 0.00 | 0.00 |  |  | [-0.01, 0.01] | 0.0% |
| **Model 2:** Positive Parenting → Phone Addiction → Total Problems |  |  |  |  |  |  |
| Total effect (c) | -0.32 | 0.04 | -8.05 | 0.00 | [-0.40, -0.24] |  |
| Direct effect (c') | -0.22 | 0.04 | -6.08 | 0.00 | [-0.30, -0.15] | 68.8% |
| Indirect effect (a × b) | -0.10 | 0.02 |  |  | [-0.15, -0.06] | 31.2% |
| Ind 1 (Parenting → Phone Addiction→ Problems) | -0.10 | 0.02 |  |  | [-0.15, -0.06] | 100% |
| Ind 2 (Parenting →Age → Problems) | 0.01 | 0.01 |  |  | [-0.05, 0.02] | 0.0% |

Note: All models controlled for child age, gender, ADHD subtype, medication status, family income, and parental education. Bootstrap samples = 5,000. CI = confidence interval.

**Table S4. Sensitivity Analysis: Alternative Model with Behavioral Problems as the Mediator between Parenting and Mobile Phone Addiction.**

| **Path/Effect** | **B** | **SE** | **t** | ***P*** | **95% CI** |
| --- | --- | --- | --- | --- | --- |
| **Model 1**: Negative Parenting → Phone Addiction → Total Problems |  |  |  |  |  |
| Total effect (c) | 0.33 | 0.04 | 8.68 | 0.00 | [0.25, 0.40] |
| Direct effect (c') | 0.19 | 0.04 | 4.36 | 0.00 | [0.10, 0.27] |
| a path (Parenting → Phone Addiction) | 0.32 | 0.03 | 9.68 | 0.00 | [0.26, 0.39] |
| b path (Phone Addiction → Problems) | 0.44 | 0.08 | 5.80 | 0.00 | [0.29, 0.59] |
| Indirect effect (a × b) | 0.14 | 0.03 |  |  | [0.09, 0.19] |
| Proportion mediated | 42.4% |  |  |  |  |
| **Model 2:** Positive Parenting → Phone Addiction → Total Problems |  |  |  |  |  |
| Total effect (c) | -0.23 | 0.05 | -4.74 | 0.00 | [-0.32, -0.13] |
| Direct effect (c') | -0.04 | 0.05 | -0.74 | 0.46 | [-0.13, 0.06] |
| a path (Parenting → Phone Addiction) | -0.32 | 0.04 | -7.96 | 0.00 | [-0.40, -0.24] |
| b path (Phone Addiction → Problems) | 0.60 | 0.08 | 8.02 | 0.00 | [0.45, 0.75] |
| Indirect effect (a × b) | -0.19 | 0.03 |  |  | [-0.27, -0.13] |
| Proportion mediated | 82.6% |  |  |  |  |

Note: All models controlled for child age, gender, ADHD subtype, medication status, family income, and parental education. Bootstrap samples = 5,000. CI = confidence interval.
